# Removal of the Thyroid Gland

**Published:** 1883-12

**Authors:** 


### Removal of the Thyroid Gland.

Extirpation of the enlarged thyroid gland has been, of late years, frequently practiced, and, on the whole, good results have been obtained, thanks chiefly to the antiseptic system of dressing. Amongst the more important recent writers on this subject may be mentioned Professor J. L. and Dr. A. Reverdin, who have published in the *Revue Medicale de la Suisse Romande* (1883, Nos. 4, 5 and 6) an account of twenty-two extirpations of goitre performed by them on twenty-one patients, twelve of whom were women. These cases are interesting, not only on account of the good results obtained, but also, and chiefly, perhaps, because Messrs. Reverdin were able to examine their patients several years after the operation, and then discovered certain remarkable alterations of the general health, which will be mentioned below, and which had, it seems, escaped the attention of other operators. The following is a brief account of Messrs. Reverdin's paper: The size of the tumors varied from that of a hen's egg to that of an adult's head. Most of the tumors were parenchymatous, with or without cysts, and occupied more or less exactly the middle line; one sent a prolongation downwards behind the sternum. In seventeen cases, total extirpation was performed; once an enucleation was done, while the remaining were cases of partial extirpation. Of the twenty-two operations, two only were followed by death, which was caused, in one case, by dyspnœa and convulsions, and, in the other, by lobular pneumonia. On an average, the patients remained only sixteen days in the hospital after the operation, and, in ten cases, union by first intention was obtained.

Amongst the accidents which may accompany or follow the operation, Messrs. Reverdin mention hemorrhage, which is best prevented by securing the thyroid arteries as soon as possible; secondary hemorrhage occurred in two cases. Dyspnœa was sometimes present before the operation, and in several cases prevented the administration of chloroform. The same complication, and also dysphonia, aphonia and dysphagia, were often noticed after the operation, but generally disappeared after a short time. In three cases, tonic convulsions of the upper extremities appeared after the operation; in one of these cases

(mentioned above), they were accompanied by a severe attack of suffocation, and soon followed by death. In five patients, three of whom were men, a remarkable alteration of the general health showed itself some months after the extirpation of the thyroid gland. There were at first weakness and coldness of the limbs, then loss of appetite, slowness of speech, diminution of the memory and progressive anæmia, accompanied, in two cases, by a peculiar œdema, most marked in the face, and very analogous to that which occurs in myxœdema. These symptoms partially disappeared after about three years. They seem to show themselves only after total extirpation, and are produced, according to Messrs. Reverdin, by a lesion of vaso-motor nerves, and by a mucoid infiltration of certain tissues, in consequence of the removal of the thyroid gland. These serious consequences, which have a remarkable analogy with cretinism on the one hand, and myxœdema on the other, must be taken into consideration in the prognosis. Nevertheless, extirpation (or, if possible, enucleation) ought to be performed, according to Messrs. Reverdin, when there are symptoms of imminent danger, such as attacks of suffocation, and also in cases of retrosternal or rapidly growing goitre, when the ordinary treatment has proved useless.—*British Medical Journal.*

